# The first description of severe anemia associated with acute kidney injury and adult minimal change disease: a case report

**DOI:** 10.1186/1752-1947-3-20

**Published:** 2009-01-23

**Authors:** Yimei Qian, Sushil K Mehandru, Nancy Gornish, Elliot Frank

**Affiliations:** 1 Department of Medicine, Jersey Shore University Medical Center, 1945 Route 33, Neptune, New Jersey 07754, USA

## Abstract

**Introduction:**

Acute kidney injury in the setting of adult minimal change disease is associated with proteinuria, hypertension and hyperlipidemia but anemia is usually absent. Renal biopsies exhibit foot process effacement as well as tubular interstitial inflammation, acute tubular necrosis or intratubular obstruction. We recently managed a patient with unique clinical and pathological features of minimal change disease, who presented with severe anemia and acute kidney injury, an association not previously reported in the literature.

**Case presentation:**

A 60-year-old Indian-American woman with a history of hypertension and diabetes mellitus for 10 years presented with progressive oliguria over 2 days. Laboratory data revealed severe hyperkalemia, azotemia, heavy proteinuria and progressively worsening anemia. Urine eosinophils were not seen. Emergent hemodialysis, erythropoietin and blood transfusion were initiated. Serologic tests for hepatitis B, hepatitis C, anti-nuclear antibodies, anti-glomerular basement membrane antibodies and anti-neutrophil cytoplasmic antibodies were negative. Complement levels (C3, C4 and CH50) were normal. Renal biopsy unexpectedly displayed 100% foot process effacement. A 24-hour urine collection detected 6.38 g of protein. Proteinuria and anemia resolved during six weeks of steroid therapy. Renal function recovered completely. No signs of relapse were observed at 8-month follow-up.

**Conclusion:**

Adult minimal change disease should be considered when a patient presents with proteinuria and severe acute kidney injury even when accompanied by severe anemia. This report adds to a growing body of literature suggesting that in addition to steroid therapy, prompt initiation of erythropoietin therapy may facilitate full recovery of renal function in acute kidney injury.

## Introduction

Acute kidney injury (ARF) in the setting of adult minimal change disease (MCD) has been well documented in the literature [1-6], but it is uncommon. Renal dysfunction is usually mild to moderate. Hemodialysis is seldom required [1,6]. MCD with ARF has been associated with older age, high blood pressure, elevated serum cholesterol, marked proteinuria and hypoalbuminemia [2,5,6]. Severe anemia has not been reported in association with MCD. Renal biopsies usually exhibit concomitant tubular interstitial inflammation, acute tubular necrosis or intratubular obstruction [2-6]. We report a case of severe oliguric ARF requiring hemodialysis, associated with significant anemia necessitating blood transfusion. The patient had normal serum lipid levels, and a renal biopsy displayed 100% foot process effacement with only proximal tubular edema, but none of the other pathologic features (tubular necrosis, interstitial nephritis, intratubular obstruction) usually found in association with ARF in this setting. Renal function recovered completely after 6 weeks of corticosteroid therapy and 8 months following presentation there was no sign of recurrence. Thus, this case is unusual in both its clinical and pathologic features and, to our knowledge, is the first reported case of MCD associated with severe anemia.

## Case presentation

A 60-year-old Indian-American woman with a history of hypertension and diabetes mellitus for 10 years presented to the emergency room with progressively worsening shortness of breath, abdominal pain, nausea, vomiting and a decreased urine output for the past 2 days. One month earlier, the patient had undergone an elective total knee replacement for osteoarthritis, and 1 week earlier the patient had been treated for urinary tract infection (UTI) with Bactrim™ (trimethoprim/sulfamethoxazole).

Hypertension and diabetes mellitus had been well controlled. The patient took diltiazem, lisinopril, metformin, glipizide and acetaminophen for knee pain. Her baseline renal function had been normal, with a serum blood urea nitrogen (BUN) level of 8 mg/dl and creatinine of 0.8 mg/dl 1 month earlier. She did not use non-steroidal anti-inflammatory drugs, whether prescribed or over the counter. The patient did not use tobacco or alcohol, nor did she have any risk factors for immunodeficiency virus (HIV) infection.

At initial presentation, her blood pressure was 148/76 mmHg, pulse 61 per minute and respiration 20 per minute. The patient was awake and oriented. There was mild bilateral ankle edema. The rest of the physical examination was unremarkable.

The serum sodium was 128 mmol/l, potassium 8.3 mmol/l, bicarbonate 19 mmol/l, BUN 52 mg/dl, and creatinine 7.0 mg/dl. The plasma albumin concentration was 1.5 g/dl. Liver function tests were normal. White blood cell count was 7200/mm^3 ^(23% lymphocytes, 9.0% monocytes, 66.1% granulocytes and 0.8% eosinophils). Hematocrit was 31.9 mg/dl. The patient's baseline hematocrit was 39.0 mg/dl. Glycosylated hemoglobin was 6.8%. Urine analysis showed 4+ proteinuria and microscopic hematuria but no crystals or casts. Lipid profile was normal: cholesterol 177 mg/dl and low-density lipoprotein (LDL) 95 mg/dl.

**Figure 1:**
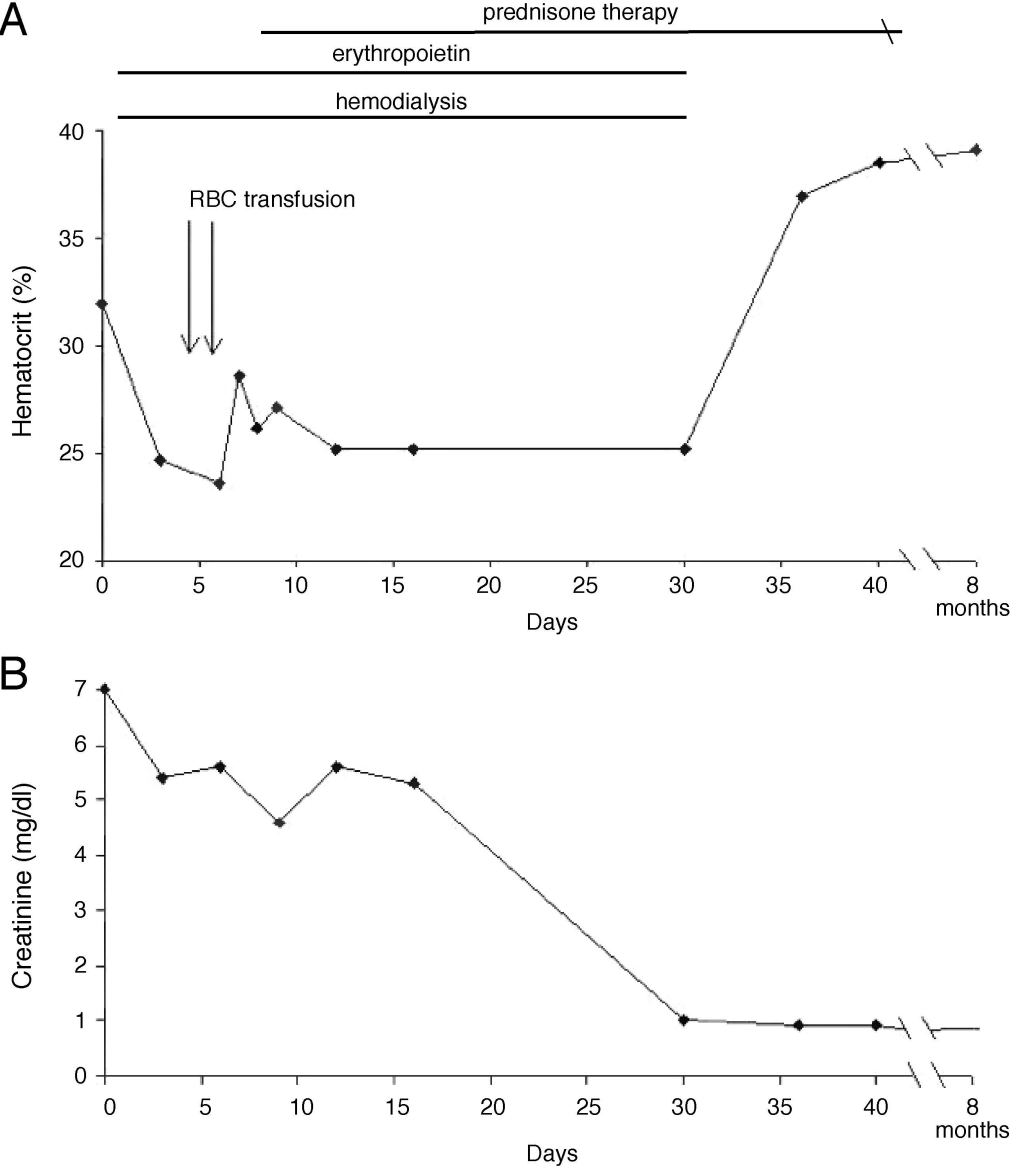
**Therapy scheme and resultant changes in hematocrit (*A*) and creatinine levels (*B*) of the patient**. Hemodialysis and erythropoietin treatments were initiated on Day one of hospitalization. Prednisone therapy was initiated on Day eight. The hematocrit percentages and creatinine levels were measured at the indicated days. The changes in hematocrit and creatinine levels correlated well with the progression of renal function.

The patient was admitted to the intensive care unit. Emergent hemodialysis was initiated (Figure 1). Erythropoietin (EPO), albumin and diuretics were also administered. A 24-hour urine collection detected 6.38 g of proteins. Erythrocyte sedimentation rate was 111 mm/hour. Urine eosinophils were negative. Abdominal computed tomography (CT) showed normal sized kidneys with no evidence of tumor, bleeding or renal obstruction. Serum protein electrophoresis revealed a decrease in total protein, albumin and gammaglobulins, but no monoclonal protein peak was detected. There was no serologic evidence of hepatitis A, B or C. Anti-nuclear antibody, anti-glomerular basement membrane (GBM) antibody and anti-neutrophil cytoplasmic antibody were not detected. Complement levels (C3, C4 and CH50) were normal.

The patient's hematocrit level continued to drop during the first week of hospitalization, reaching a nadir of 23.6 mg/dl and requiring packed red blood cell transfusion on two occasions (Figure 1A). There were no schistocytes on peripheral smear and bilirubin was normal. Fecal occult blood test remained negative and there was no other evidence of bleeding. The patient received intravenous fluids only transiently (Days two and three while hospitallized at 60 cc per hour), and the patient's weight during this period remained stable (89.7 kg on admission and 89.4 kg on Day four in hospital) making hemodilution unlikely.

**Figure 2:**
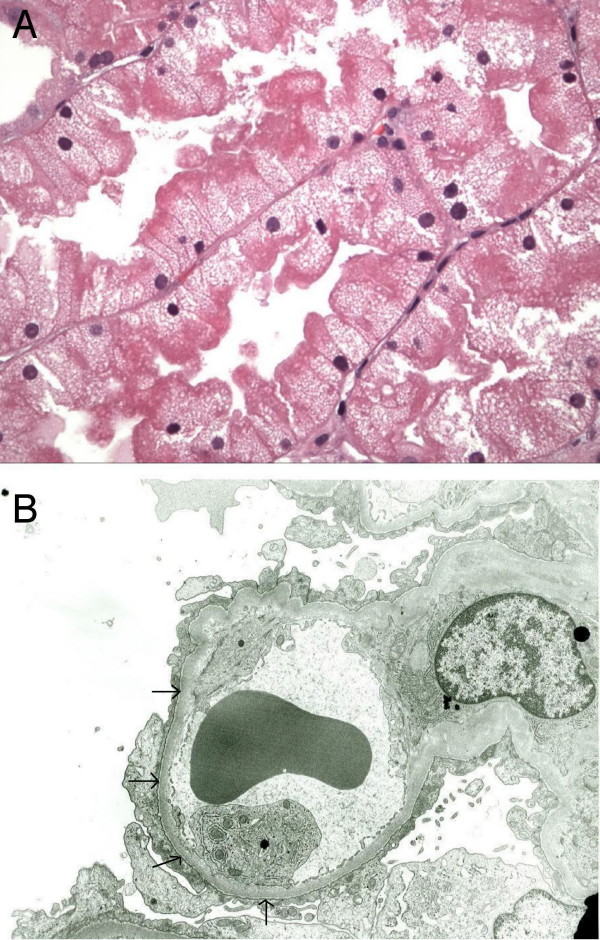
**Histopathologic examination of renal biopsy from the patient**. (***A***) Light microscopy: image showing proximal tubules with marked cytoplasmic swelling and vacuolization. (***B***) Electron microscopy: image showing glomerular basement membrane effacement (arrows). The basement membrane shows 100% foot process effacement without any electron-dense deposit.

A renal biopsy was performed. Examination by light microscopy showed 14 glomeruli, all of which appeared normal. Cytoplasmic swelling was noted in the proximal tubules (Figure 2A). Electron microscopy revealed lipid droplets, extensive microvillous transformation in visceral epithelial cells and 100% foot process effacement (Figure 2B). The glomerular basement membrane was of normal thickness and contour. No electron dense deposits were seen. Immunofluorescent staining for immunoglobulin G, M and A, C3, C1q, fibrinogen, albumin, kappa and lambda showed no immune complex deposition. A diagnosis of MCD with ARF was established.

Prednisone 60 mg/day was initiated (Figure 1). Within 6 weeks, the patient's urine volume and renal function returned to normal. Resolution of anemia followed the renal recovery. Hemodialysis and EPO were discontinued on Day 30. The steroid dosage was tapered after renal function recovered completely and stopped entirely, following 5 months of treatment. At 8-month follow-up, the patient's creatinine was 0.8 mg/dl and there was no proteinuria.

## Discussion

Oliguric acute kidney injury occurs infrequently in adult MCD [1,2]. The usual clinical setting is elderly patients with anasarca, massive proteinuria, low serum albumin concentration, hypertension and hypercholesterolemia [1-6]. Renal insufficiency occurs with no apparent precipitating event, generally soon after the onset of the nephrotic syndrome. The reduction of glomerular filtration rate is generally less than 50% [2-6]. In a small proportion of patients, however, the functional impairment is severe and irreversible, requiring hemodialysis [1,6]. Although anemia has been reported as a presentation in severe ARF with other renal diseases [7], it has not been reported as a presentation in ARF with adult MCD. In our patient, the creatinine was 7.0 mg/dl, hematocrit 31.9 mg/dl, cholesterol 177 mg/dl and LDL 95 mg/dl on presentation. This clinical constellation – acute azotemia, oliguric-anuric ARF, rapidly declining hematocrit and a completely normal lipid profile – led us to explore a variety of potential causative or contributory factors including hypovolemia, autoimmune diseases, infectious diseases, drug toxicity, allergic reaction and renal obstruction. However, clinical assessment, laboratory evaluation and imaging studies excluded these etiologies. Surprisingly, the renal biopsy identified MCD and only proximal tubular interstitial edema, so-called "osmotic nephrosis" with patent lumina (Figure 2). Osmotic nephrosis describes a pattern of tubular injury most commonly seen with exposure to non-metabolizable macromolecules such as mannitol, radiocontrast media or dextran. In our case, it is unlikely that dye exposure played a significant role since the patient developed ARF prior to the contrast administration, and the patient had hemodialysis shortly after the dye exposure. Unlike the scenario in the majority of cases of MCD with severe ARF, our patient did not have any evidence of concomitant acute tubular necrosis, interstitial nephritis or significant intratubular obstruction.

Thaysen et al. [7] described severe anemia associated with ARF in patients with acute crescentic glomerulonephritis or bilateral tubular necrosis, the types of renal disease frequently accompanied by severe acute kidney injury. A dramatic drop in hemoglobin concentration usually occurred several days after the onset of ARF [7]. Restoration of red blood cell mass took place only after significant improvement of renal function. Most of these patients had only partial renal recovery or remained on chronic dialysis. The recovery of anemia was sluggish and normalization of hemoglobin levels was observed only in patients with substantial renal recovery.

To date, anemia has not been reported in ARF associated with adult MCD, although this may be due to the paucity of cases of severe ARF from MCD. Our patient presented with severe ARF and moderately reduced hemoglobin, which continued to drop dramatically during the first several days of hospitalization. Although our patient had microscopic hematuria during the oliguric phase, a finding seen in about one-third of patients with MCD [6], it is not associated with, nor can it account for, severe anemia. Investigation for evidence of overt bleeding or hemolysis was negative, suggesting that anemia was due to diminished EPO levels associated with ARF. Indeed, the hematocrit stabilized four days after initiating EPO therapy and rose to normal level following full recovery of renal function (Figure 1A). This presentation closely resembles the characteristics of the anemia observed in the cases with acute crescentic glomerulonephritis or bilateral tubular necrosis.

One intriguing recent observation is the possible tissue-protective effect of EPO. *In vitro *and animal studies have suggested that EPO might promote renal recovery and decrease mortality in ARF [8,9]. The EPO receptor is present in the glomerulus, mesangial and tubular epithelial cells in the kidney. EPO or its analogs administered before or immediately after the onset of renal injury reduce tubular damage, enhance tubular epithelial cell regeneration and promote renal functional recovery [8,9]. There is a paucity of human data evaluating this issue, limited to a few series with encouraging results. Unfortunately, in the clinical setting, most cases of acute kidney injury are not identified until some time after renal functional deterioration has occurred and significant anemia emerges several days later [7], decreasing the likelihood that EPO would be administered early in the course of ARF. Moreover, there is no consensus on the use of EPO in this setting. Most physicians prescribe EPO for patients with anemia who had a prior diagnosis of chronic kidney injury rather than for those with ARF [10]. This may explain why a recent retrospective study of 187 critically ill patients with ARF failed to show a beneficial effect of EPO in ARF [10]. In this study, EPO was administered at any time during the first 14 days of the initiation of renal replacement therapy. In contrast, in our case, high-dose EPO was initiated at the onset of ARF and 4 days before the significant drop in hematocrit (Figure 1A), a scenario more closely resembling the experimental models [8]. Renal function recovered completely without relapse in spite of the severity of ARF at the initial presentation. Although spontaneous or steroid-induced remission of MCD might account for all the improvement, the unusual and dramatic recovery raises the possibility that the early use of EPO may have facilitated the recovery of renal function.

In combination with the experimental data, our findings then support the notion that EPO, administered early in the course of ARF, may have salutary effects beyond its effects on hematopoiesis, a hypothesis that should be further explored.

## Conclusion

Adult MCD may present with severe ARF, significant anemia and a normal lipid profile. Severe anemia associated with EPO deficiency may occur abruptly in this setting. Erythropoietin may attenuate renal injury and facilitate renal recovery in addition to its function on hematopoiesis if treatment is initiated early. Regardless of the age at onset or severity of ARF, renal and hemopoietic function can recover completely

## Abbreviations

ARF: acute kidney injury; MCD: minimal change disease; UTI: urinary tract infection; BUN: blood urea nitrogen; HIV: immunodeficiency virus; LDL: low-density lipoprotein; EPO: erythropoietin; CT: computer tomography; GBM: glomerular basement membrane

## Competing interests

The authors declare that they have no competing interests.

## Authors' contributions

YQ reviewed the literature and drafted the paper. SM interpreted patient data, identified the unusual nature of the case, advised on content of the paper and critically reviewed the content of the paper. NG provided clinical information and critically reviewed the content of the paper. EF interpreted patient data and the literature and critically reviewed the content of the paper. All authors read and approved the final manuscript.

## References

[B1] Cameron MA Peri U Rogers TE Moe OW Minimal change disease with acute renal failure: a case against the nephrosarca hypothesis Nephrol Dial Transplant 2004 19 10 2642 2646 10.1093/ndt/gfh332 15388821

[B2] Chen CL Fang HC Chou KJ Lee JC Lee PT Chung HM Wang JS Increased endothelin 1 expression in adult-onset minimal change nephropathy with acute renal failure Am J Kidney Dis 2005 45 5 818 825 10.1053/j.ajkd.2005.02.007 15861346

[B3] Jennette JC Falk RJ Adult minimal change glomerulopathy with acute renal failure Am J Kidney Dis 1990 16 5 432 437 2239933 10.1016/s0272-6386(12)80055-2

[B4] Lowenstein J Schacht RG Baldwin DS Renal failure in minimal change nephrotic syndrome Am J Med 1981 70 2 227 233 10.1016/0002-9343(81)90754-3 7468609

[B5] Mak SK Short CD Mallick NP Long-term outcome of adult-onset minimal-change nephropathy Nephrol Dial Transplant 1996 11 11 2192 2201 8941578 10.1093/oxfordjournals.ndt.a027136

[B6] Waldman M Crew RJ Valeri A Busch J Stokes B Markowitz G D'Agati V Appel G Adult minimal-change disease: clinical characteristics, treatment, and outcomes Clin J Am Soc Nephrol 2007 2 3 445 453 10.2215/CJN.03531006 17699450

[B7] Thaysen JH Nielsen OJ Brandi L Szpirt W Erythropoietin deficiency in acute crescentic glomerulonephritis and in total bilateral renal cortical necrosis J Intern Med 1991 229 4 363 369 2026990 10.1111/j.1365-2796.1991.tb00360.x

[B8] Johnson DW Pat B Vesey DA Guan Z Endre Z Gobe GC Delayed administration of darbepoetin or erythropoietin protects against ischemic acute renal injury and failure Kidney Int 2006 69 10 1806 1813 10.1038/sj.ki.5000356 16598197

[B9] Spandou E Tsouchnikas I Karkavelas G Dounousi E Simeonidou C Guiba-Tziampiri O Tsakiris D Erythropoietin attenuates renal injury in experimental acute renal failure ischaemic/reperfusion model Nephrol Dial Transplant 2006 21 2 330 336 10.1093/ndt/gfi177 16221709

[B10] Park J Gage BF Vijayan A Use of EPO in critically ill patients with acute renal failure requiring renal replacement therapy Am J Kidney Dis 2005 46 5 791 798 10.1053/j.ajkd.2005.07.034 16253718

